# Ultrasound-Guided Synovial Biopsy in Undifferentiated Monoarthritis: A Case Series From Bangladesh

**DOI:** 10.7759/cureus.86870

**Published:** 2025-06-27

**Authors:** Mohammad Abul Kalam Azad, M Masudul Hassan, Minhaj Rahim Choudhury, Ariful Islam, Abul Khair Ahmedullah

**Affiliations:** 1 Rheumatology, Bangladesh Medical University, Dhaka, BGD; 2 Rheumatology, Bangabandhu Sheikh Mujib (BSM) Medical University, Dhaka, BGD

**Keywords:** genexpert assay, synovial biopsy, synovial fluid, tuberculous arthritis, usg guided synovial biopsy

## Abstract

Ultrasound-guided synovial biopsy (USG-SB) is a minimally invasive technique useful in diagnosing joint tuberculosis, particularly when conventional methods fail. However, its role in diagnosing tubercular arthritis in Bangladesh remains underexplored. This study presents a case series of five patients with monoarthritis - three with ankle involvement and two with knee joint tuberculosis - evaluated using USG-SB or aspiration. The procedures were performed using a high-resolution musculoskeletal ultrasound (14-6 MHz probe, Mindray, Shenzhen, China). Synovial tissue samples were analyzed via histopathology and GeneXpert (Cepheid, Sunnyvale, California, US) testing, while synovial fluid was examined via cytology, acid-fast bacilli (AFB) staining, and GeneXpert. Tuberculosis was confirmed histologically in three cases, GeneXpert was positive in two, and AFB staining was positive in one. These findings support USG-SB as a safe, effective, and minimally invasive diagnostic option for tubercular arthritis, particularly in resource-limited settings like Bangladesh.

## Introduction

Synovial biopsy (SB) is often necessary when the clinical presentation is nonspecific and a definitive diagnosis is needed. Traditionally, a synovial biopsy is performed via open surgery or arthroscopy, both of which are invasive methods [1]. In contrast, ultrasound-guided synovial biopsy (USG-guided SB) is a less invasive technique that offers improved diagnostic accuracy. Ultrasound provides both dynamic and static visualization of joint structures, facilitating precise needle placement and reducing the risk of complications. This makes it a safe and effective method for obtaining synovial tissue for histopathological examination, including molecular tests such as GeneXpert (Cepheid, Sunnyvale, California, US).

In 2024, an estimated 10.8 million people were infected with *Mycobacterium tuberculosis*, according to the World Health Organization (WHO) [2]. Tuberculous arthritis is the second most common form of extrapulmonary tuberculosis and poses significant diagnostic challenges due to its deep joint location, nonspecific clinical presentation, and resemblance to other types of arthritis [3]. As a result, diagnosis is often delayed.

At the Department of Rheumatology, Bangladesh Medical University (BMU), synovial biopsies were performed using a Mindray ultrasound machine (Shenzhen, China) with a 14-6 MHz linear probe to investigate cases of monoarthritis. All procedures were conducted under strict aseptic conditions following written informed consent. Patients were monitored for 72 hours post-procedure, and no complications were observed.

The differential diagnoses for tuberculous arthritis include traumatic arthritis, pyogenic arthritis, rheumatoid arthritis, psoriatic arthropathy, sarcoidosis, and neoplastic conditions. In this case series, five patients underwent USG-guided synovial biopsy. Among them, three were male and two were female, with a mean age of 44.4 years. Four patients were from low socioeconomic backgrounds. None had a history of pulmonary tuberculosis, although two patients were diabetic, and one had a history of spondyloarthritis.

## Case presentation

Case 1: tubercular arthritis of the right ankle joint

A 50-year-old Bangladeshi male, a cultivator by occupation and known diabetic, was admitted to the Department of Rheumatology at Bangladesh Medical University with a five-month history of pain and swelling in the left ankle joint.

The patient was in good health approximately eight years earlier while residing abroad. At that time, he developed low back pain (LBP) of insidious onset, which gradually worsened and was associated with significant morning stiffness. The pain was moderate in severity and intermittently relieved with non-steroidal anti-inflammatory drugs (NSAIDs). This episode of LBP resolved spontaneously two years ago without further recurrence.

Five months prior to admission, he developed gradual-onset pain and swelling in the left ankle joint. The symptoms progressively worsened, were mechanical in nature, and of moderate intensity. He initially consulted a local physician and received an intra-articular triamcinolone injection, which provided minimal relief. Subsequent treatment with sulphasalazine and systemic corticosteroids also failed to produce significant improvement.

The patient denied any history of painful red eyes and psoriatic skin lesions. He also reported no systemic symptoms such as anorexia, weight loss, low-grade fever, or night sweats. There was no prior history of tuberculosis or known exposure to a smear-positive TB patient. He is an ex-smoker and has received the COVID-19 vaccine.

On physical examination, the left ankle joint was tender, swollen, and exhibited restricted range of motion. No abnormalities were noted on systemic examination.

Given his previous history of inflammatory back pain, possible enthesitis, and current peripheral joint involvement, an initial diagnosis of spondyloarthropathy was considered. However, the presence of isolated monoarthritis of the ankle, particularly in the absence of other typical features of spondyloarthropathy, raised the suspicion of tuberculous arthritis, despite the lack of classical systemic TB signs.

**Figure 1:**
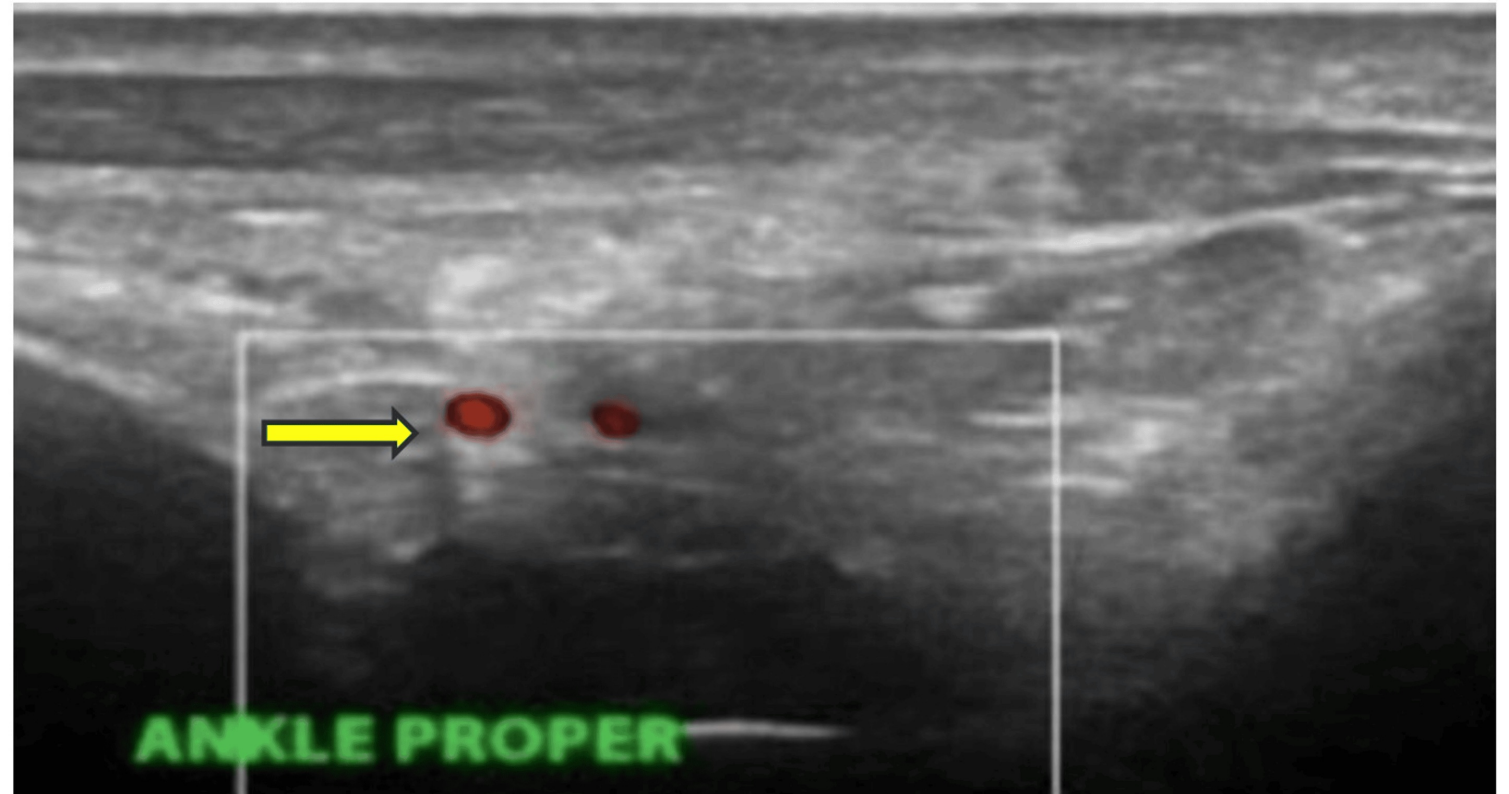
Ultrasound findings of the left ankle anterior longitudinal view showing synovial hypertrophy (yellow arrow) with a grade 2 Doppler signal

**Figure 2:**
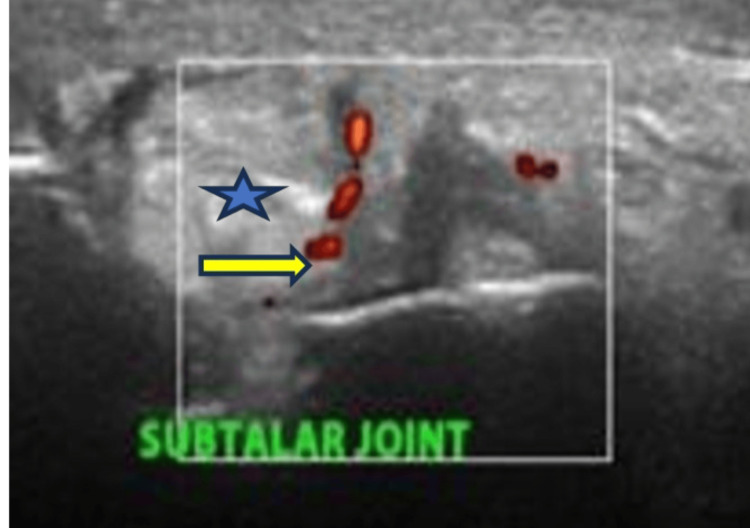
Ultrasound findings of the subtalar joint on the medial transverse view showing synovial hypertrophy with a grade 2 Doppler signal The yellow arrow indicates a grade 2 Doppler signal; the green star indicates synovial hypertrophy.

**Figure 3:**
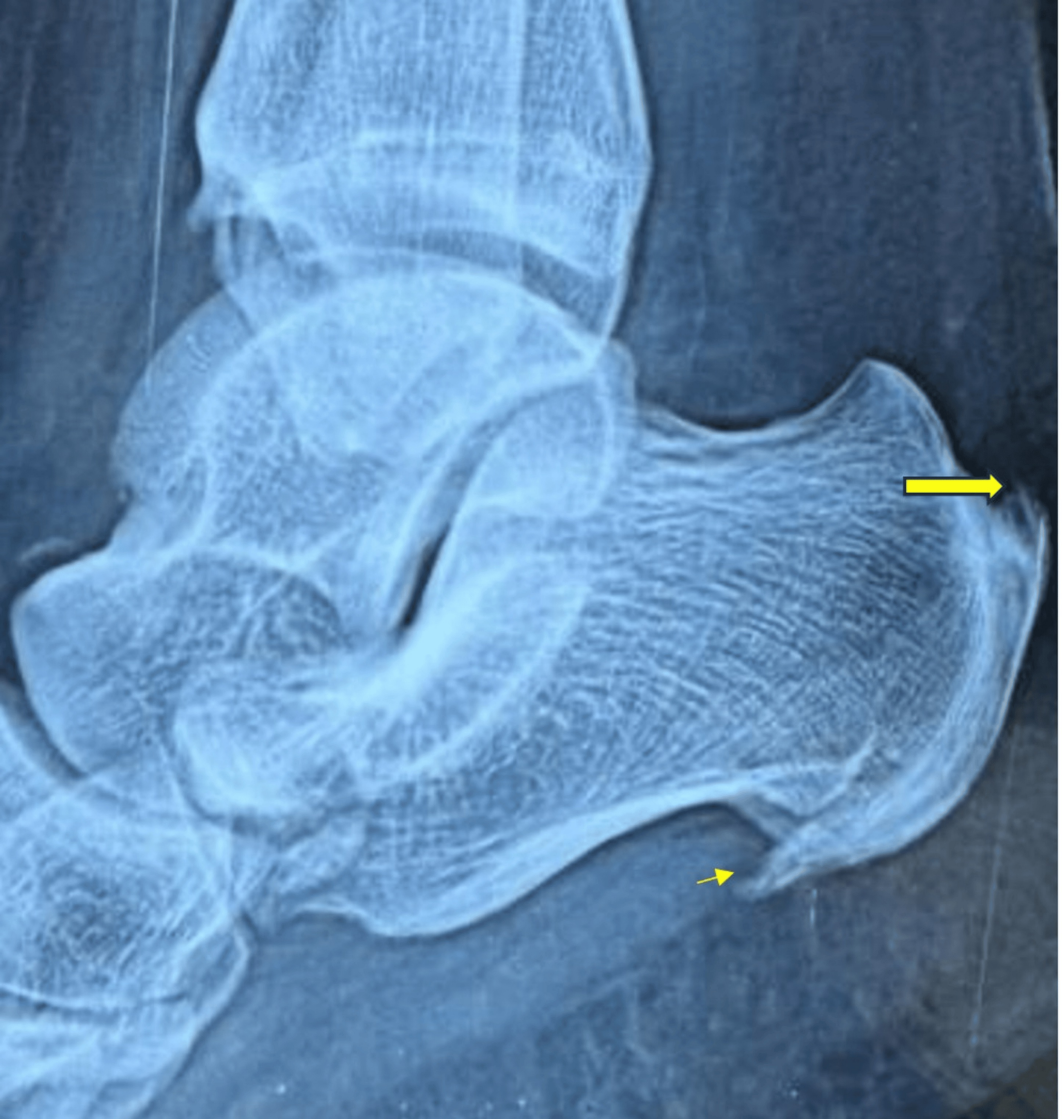
X-ray ankle shows a retrocalcaneal spur The large arrow indicates the retrocalcaneal spur, and the small arrow indicates the plantar spur.

Due to the diagnostic uncertainty, a synovial biopsy of the affected ankle joint was performed. Histopathological analysis confirmed the diagnosis of tuberculous arthritis. Musculoskeletal ultrasound of the left ankle and subtalar joints revealed features consistent with inflammatory arthritis (Figures 1, 2). An X-ray of the ankle showed the presence of a retrocalcaneal spur (Figure 3).

The patient was started on standard four-drug anti-tuberculosis therapy (ATT), consisting of isoniazid, rifampicin, pyrazinamide, and ethambutol. His condition improved significantly following six months of treatment.

**Figure 4:**
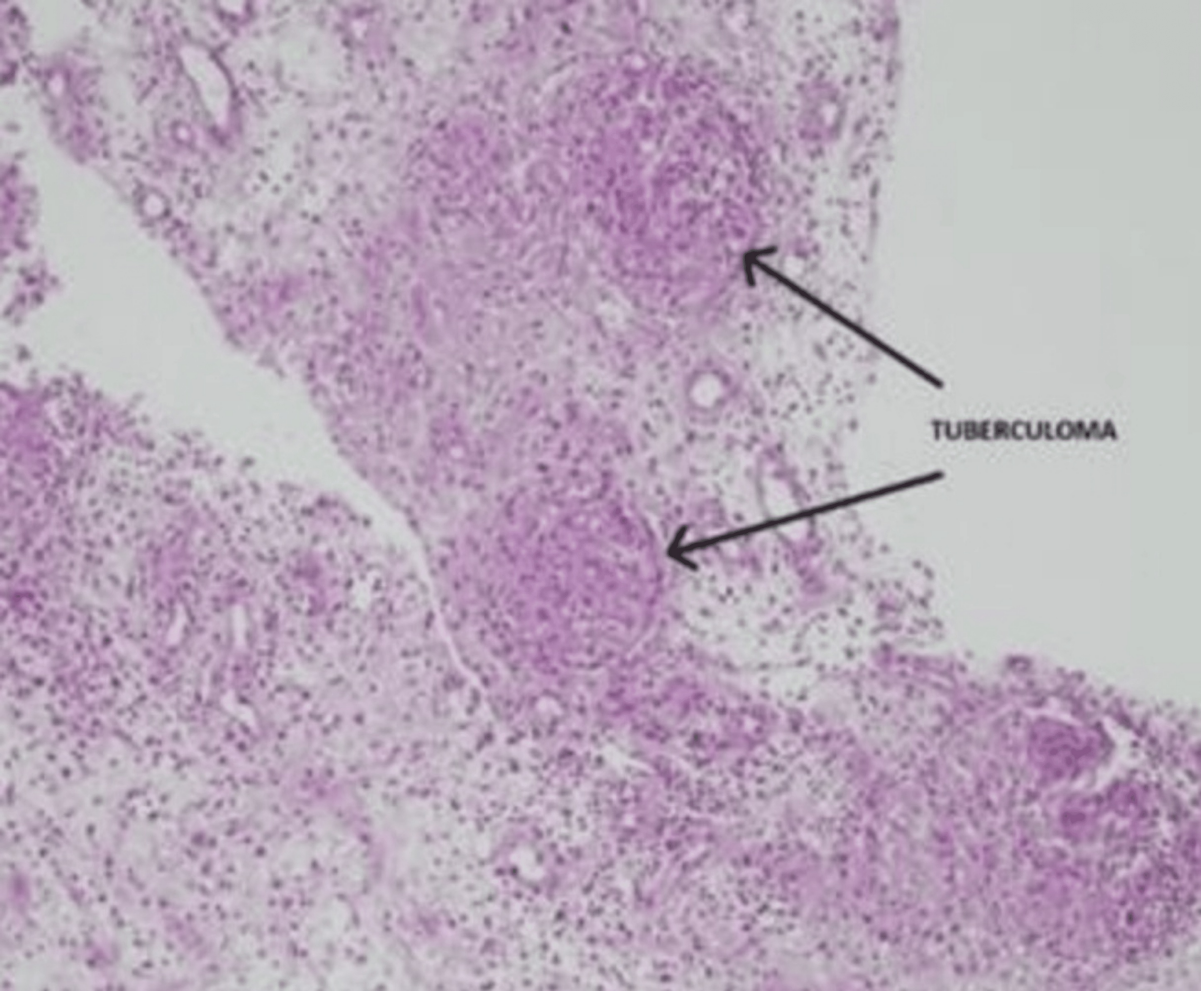
Microscopic histopathological examination of the synovial tissue shows multiple granulomas (staining by H&E) The black arrow indicates multiple granulomas (tuberculomas) with chronic inflammatory cells. Photograph courtesy of the Department of Pathology, Bangladesh Medical University (BMU).

A synovial biopsy from the left ankle joint through the lateral approach was done for a histopathological examination with GeneXpert. It shows a specimen that consists of four gray-white areas of tissue. The largest one measures 0.6×0.3 cm, and the smallest one measures 0.3×0.2 cm. It reveals multiple epithelioid-type granulomas surrounded by lymphocytes, histologically consistent with tuberculosis in Figure 4.

Case 2: tubercular arthritis of the right ankle joint

A 17-year-old girl was admitted to a tertiary care hospital in Bangladesh with a one-month history of fever and a seven-day history of right ankle pain and swelling. Her fever was characterized by an evening rise in temperature. On admission, her right ankle joint appeared globally swollen and warm, particularly below the lateral malleolus, with restricted movement in all directions. The pain worsened with weight-bearing.

Despite the absence of joint aspiration, she was initially diagnosed with septic arthritis and treated empirically with intravenous ceftriaxone and oral flucloxacillin for 14 days. Her symptoms partially improved, and she was discharged.

Three weeks after discharge, the pain and swelling in her ankle recurred. She consulted a physician and underwent further investigations. Laboratory findings included CRP 3 mg/L (normal <6 mg/L), negative rheumatoid factor, and negative anti-CCP antibodies.

She was then prescribed oral methylprednisolone 16 mg daily for 3 weeks, along with 3 doses of benzathine penicillin and sulfasalazine 2 g/day. However, there was poor clinical response, and there was no conclusive evidence of rheumatic fever or spondyloarthropathy. The patient could not recall the exact duration of this treatment regimen.

**Figure 5:**
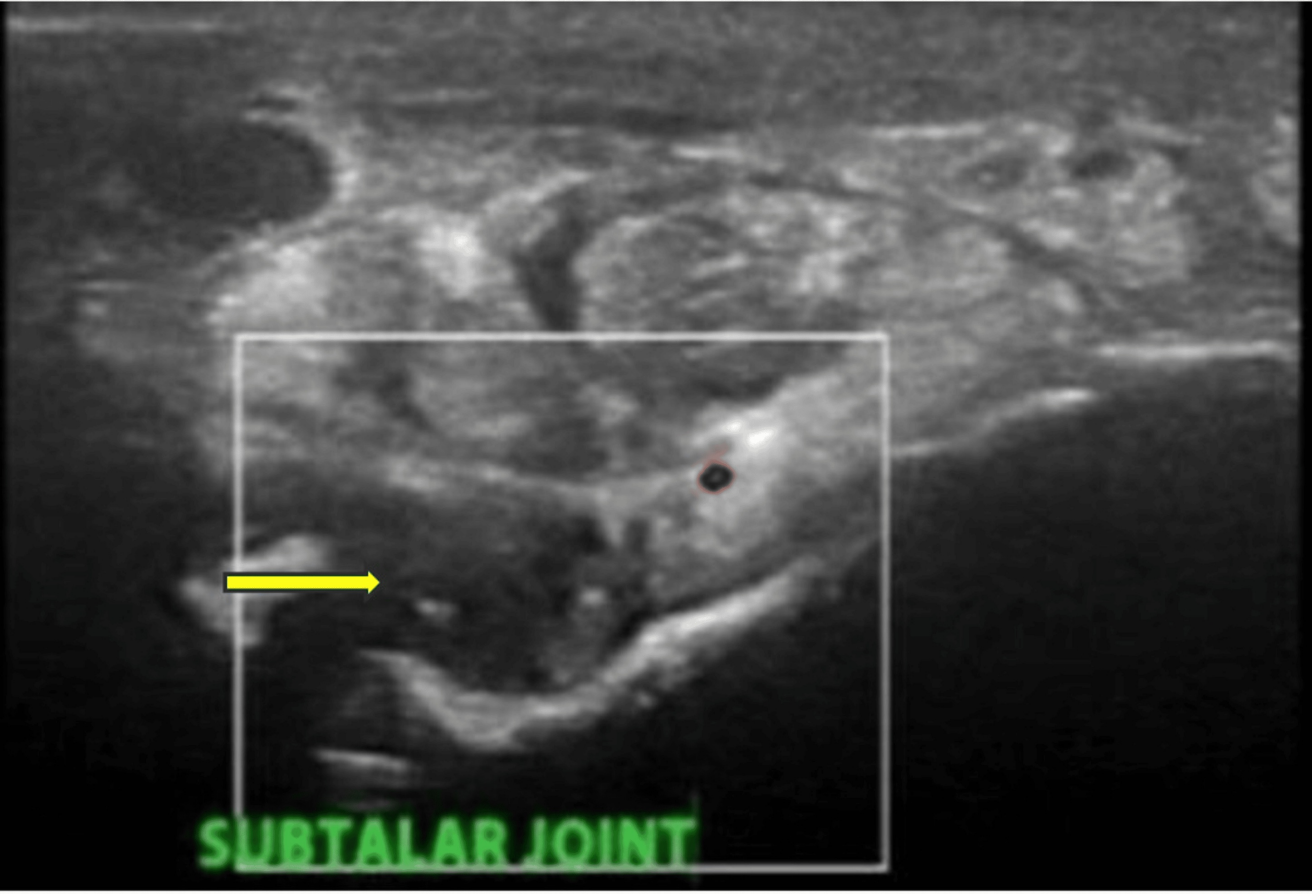
Ultrasonography (USG) of subtalar joint showing synovial hypertrophy with grade 1 Doppler The yellow arrow indicates a hypoechoic area in the subtalar joint with grade 1 Doppler.

A musculoskeletal ultrasound (Mindray) of the ankle revealed moderate synovial hypertrophy with a grade 1 Doppler signal (Figure 5), suggestive of ongoing synovitis.

**Figure 6:**
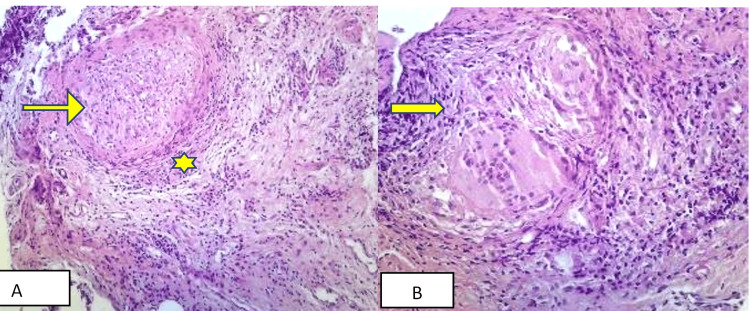
Microscopic examination of the histopathological finding of synovial tissue showing caseous necrosis and epithelioid-type granulomas surrounded by lymphocytes The arrow in (A) indicates caseous necrosis, the yellow star indicates acute and chronic inflammatory cells, and the arrow in (B) indicates epithelioid-type granulomas surrounded by lymphocytes.

She took sulfasalazine 2 g daily for another three months without significant improvement. In the next follow-up visit, she was planned for an ultrasound-guided core needle biopsy of the synovium, and histopathology showed caseation necrosis and granuloma, composed of epithelioid cells and infiltration of acute and chronic inflammatory cells in Figures 6A-6B.

Combination anti-tubercular therapy was started. Her joint pain improved gradually over the next month, and she could walk alone with mild pain.

Case 3: tubercular arthritis of the left ankle joint

**Figure 7:**
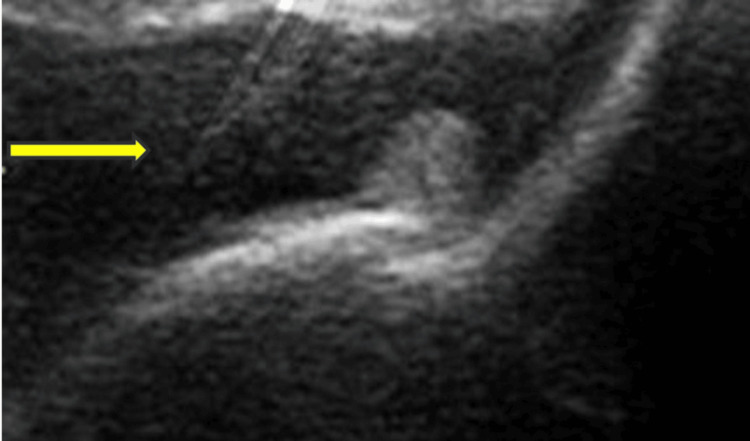
Ultrasonography (USG) of the ankle joint, anterior longitudinal view (synovial tissue) The yellow arrow indicates an anechoic area in the ankle joint.

A 50-year-old man, a cultivator, presented with complaints of pain in the left ankle joint for six months. He was also suffering from low-grade fever with significant weight loss. He has also been suffering from diabetes for one year without any complications. Routine blood test, including Hb, total count, and differentials, was normal, ESR was 50 mm in the first hour, CRP was 47 mg/dl, and the Mantoux test (MT) was 8 mm in the first hour. The chest X-ray posterior-anterior view was normal. X-ray of the left ankle joint showed a calcaneal spur, and USG showed ankle joint hypertrophy with fluid (Figure 7).

**Figure 8:**
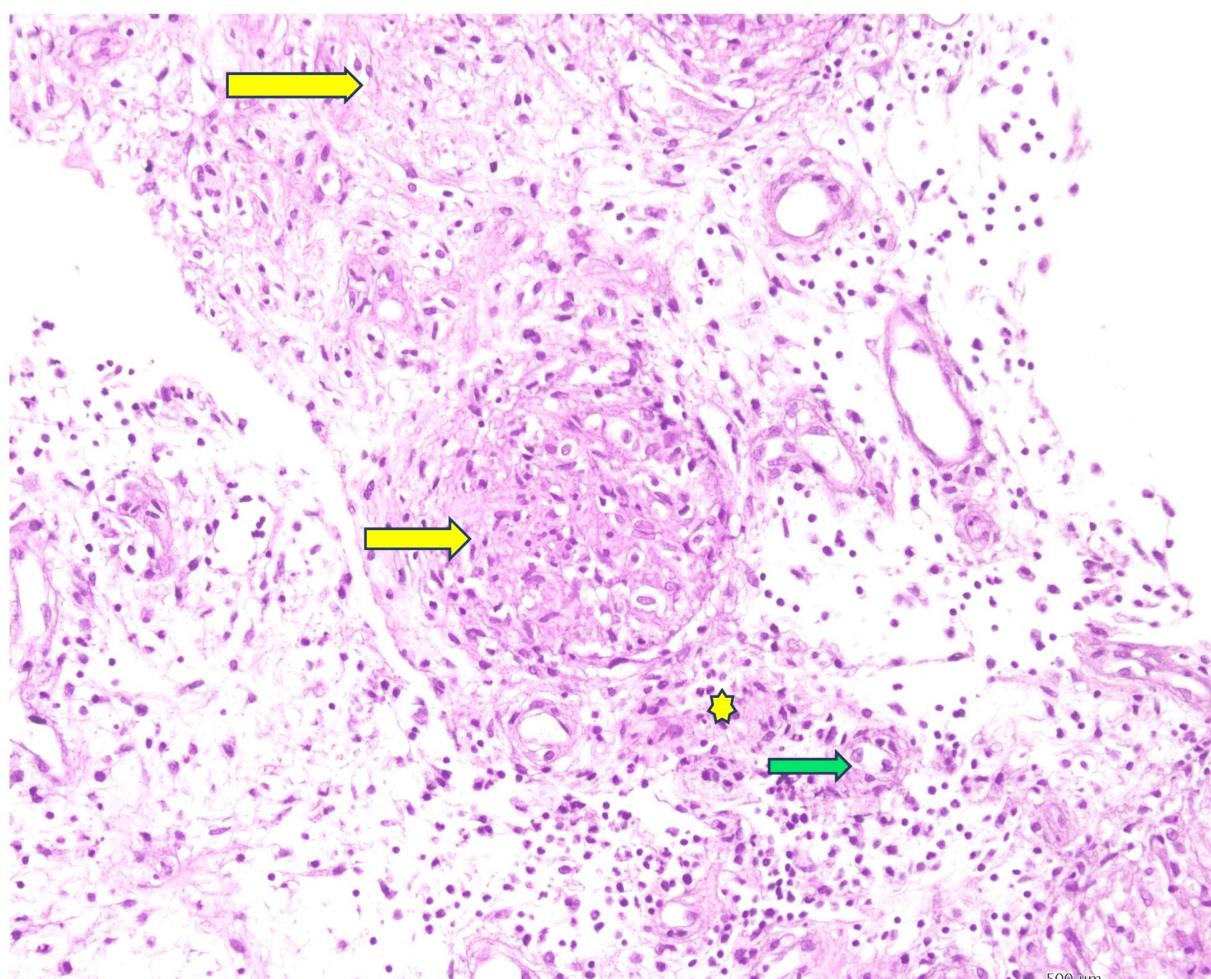
Microscopic examination of the histopathology of the synovial tissue showing multiple granulomas, composed of epithelioid cells, and infiltration of acute and chronic inflammatory cells The yellow arrow indicates granuloma, the green arrow indicates epithelioid-type giant cells, and the yellow star indicates acute and chronic inflammatory cells.

A synovial fluid study was not done. The synovial biopsy shows caseous necrosis with an epithelioid-type granuloma in Figure 8, with negative GeneXpert.

Case 4: tubercular arthritis of the right knee joint

A 45-year-old male, a cultivator, presented with a 1.5-year history of right knee pain and recurrent swelling. The symptoms began insidiously and gradually worsened over time. The pain was mild to moderate in intensity, associated with morning stiffness lasting around 30 minutes, and was aggravated by walking. It showed partial relief with analgesics. Throughout the course of the illness, the patient experienced intermittent swelling of the right knee, which typically responded to medication.

**Figure 9:**
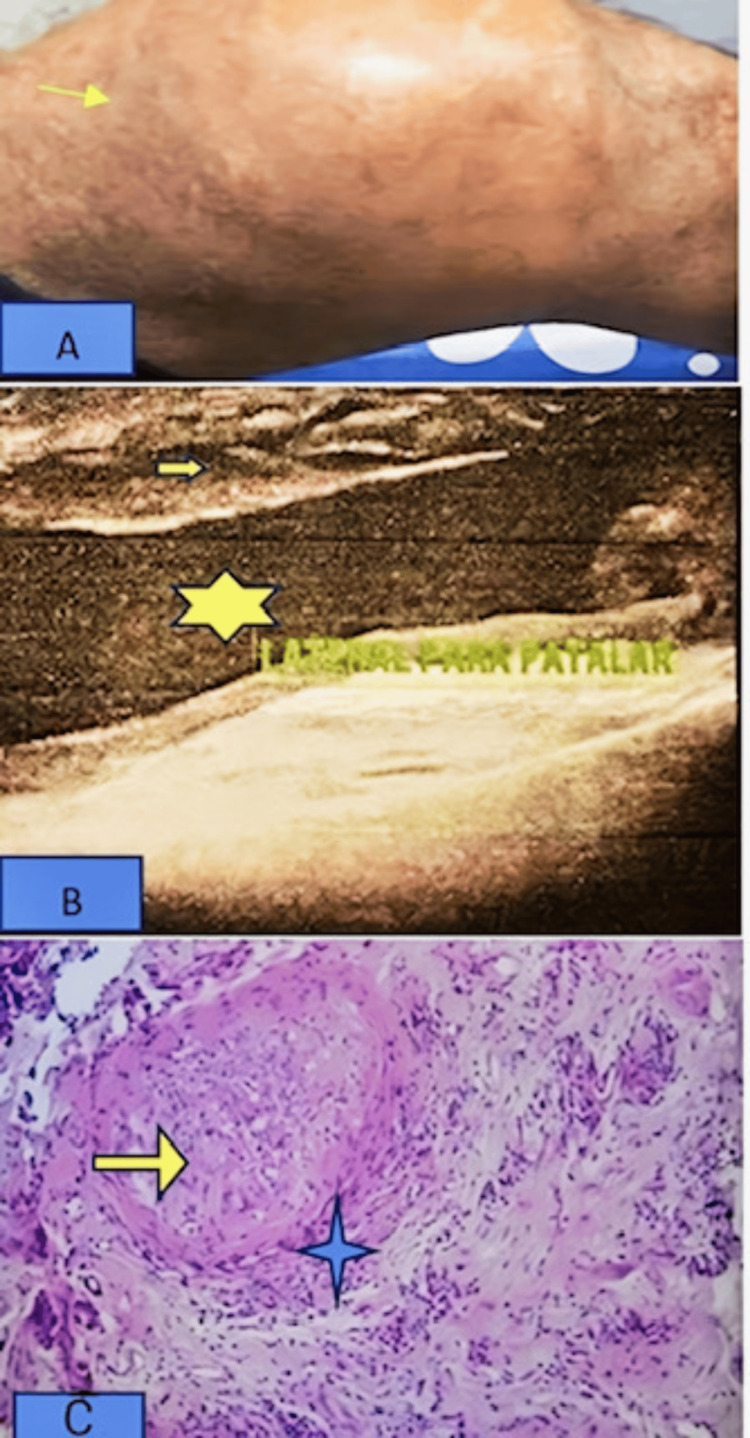
Anatomic, ultrasonographic, and microscopic findings of the knee joint (A) The arrow indicates a grossly swollen knee joint, (B) ultrasonography (USG) finding: the arrow indicates synovial hypertrophy, the yellow star indicates fluid in the suprapatellar region, (C) microscopic examination (histopathology): the arrow indicates caseation necrosis with a granuloma, with the * indicating chronic inflammatory cells

However, during the 15 days prior to admission, both pain and swelling significantly increased, to the extent that he required assistance to walk. He denied symptoms suggestive of systemic inflammatory disorders, such as inflammatory back pain, heel pain, uveitis, bloody diarrhea, psoriasis, or dactylitis. There was also no history of fever, cough, hemoptysis, contact with smear-positive tuberculosis patients, or any personal or family history of tuberculosis. The patient did report a loss of appetite and unintentional weight loss of approximately 4 kg over the course of his illness. On physical examination, the right knee was diffusely swollen, with visible wasting of the thigh muscles (Figure 9A). The joint was warm to the touch and exhibited grade 3 tenderness along the joint line. The patellar tap was positive. Both active and passive range of motion were severely restricted and painful.

Ultrasound imaging of the right knee showed significant synovial hypertrophy and joint effusion in the suprapatellar region (Figure 9B). Aspiration of the knee joint yielded turbid fluid. Ziehl-Neelsen staining of the synovial fluid was positive for acid-fast bacilli, and GeneXpert testing confirmed the presence of *Mycobacterium tuberculosis* (MTB) with sensitivity to rifampicin. The synovial fluid had a markedly elevated white cell count of 120,000 cells/mm³, predominantly consisting of polymorphonuclear leukocytes. A synovial biopsy revealed granulomatous inflammation with infiltration of chronic inflammatory cells, findings consistent with tuberculous arthritis (Figure 9C).

Based on these findings, the patient was diagnosed with tubercular arthritis of the right knee joint. He was promptly initiated on anti-tubercular medication.

Case 5: tubercular arthritis of the left knee joint

**Figure 10:**
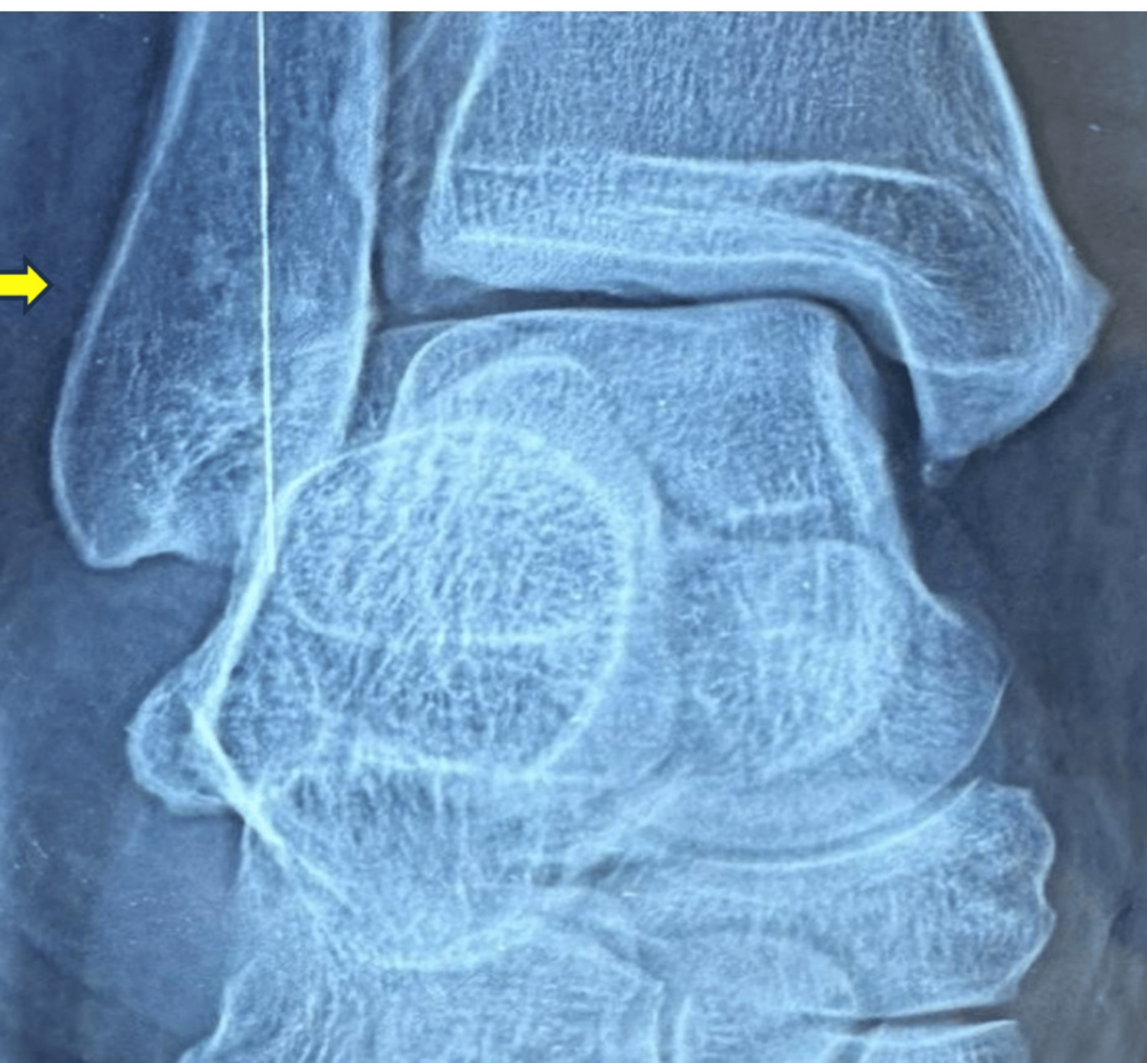
X-ray of the ankle joint shows soft tissue swelling The arrow indicates soft tissue swelling on the lateral side of the ankle.

A 60-year-old businessman, a diabetic, was admitted to the BMU with complaints of pain and swelling in the left knee joint for six months. The pain was inflammatory and did not respond to two intra-articular steroid injections. There was no history of trauma to joints, fever, weight loss, malaise, contact with TB patients, or history of TB. On query, he gave a history of inflammatory oligoarthritis four months ago, for which he was labeled as a case of peripheral spondyloarthropathy and treated with nonsteroidal anti-inflammatory drugs (NSAIDs) and sulfasalazine. His symptoms improved with these medications, but pain in the left knee joint persisted. There was no history of lower back pain, painful red eye, swelling of digits, skin lesions, or urethral discharge. On examination, vitals were within normal limits, the left knee joint was swollen with grade 2 tenderness, raised local temperature, and fixed flexion deformity. There was swelling in the back of the left lower leg, which extended from the back of the knee joint to the upper two-thirds of the calf muscle, firm in consistency, with raised temperature. An X-ray of the ankle joint shows soft tissue swelling in the lateral malleolus in Figure 10.

**Figure 11:**
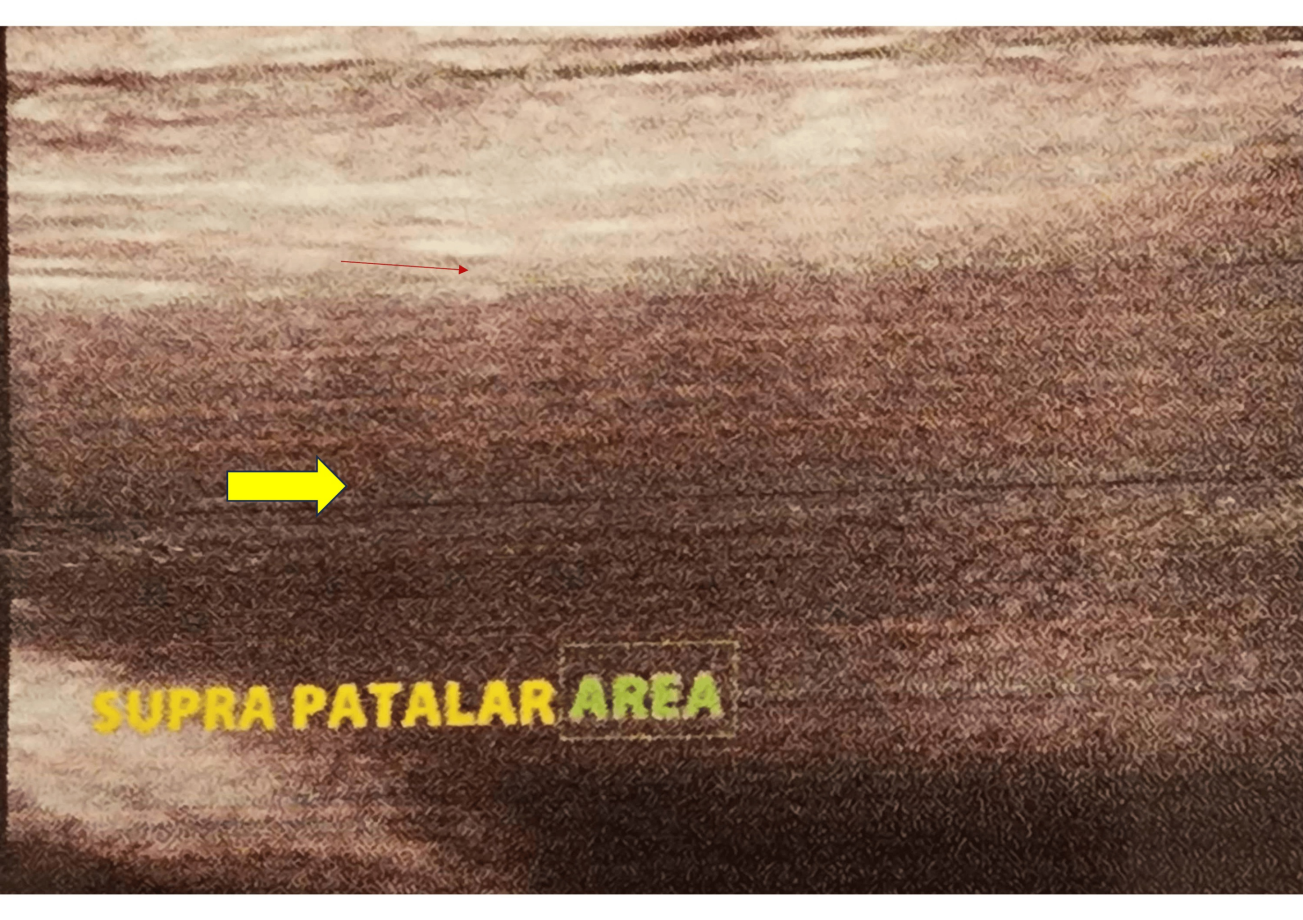
Ultrasonography (USG) of the knee joint showing fluid with synovial hypertrophy in the suprapatellar region The thick arrow indicates an anechoic area, and the thin arrow indicates synovial hypertrophy.

**Figure 12:**
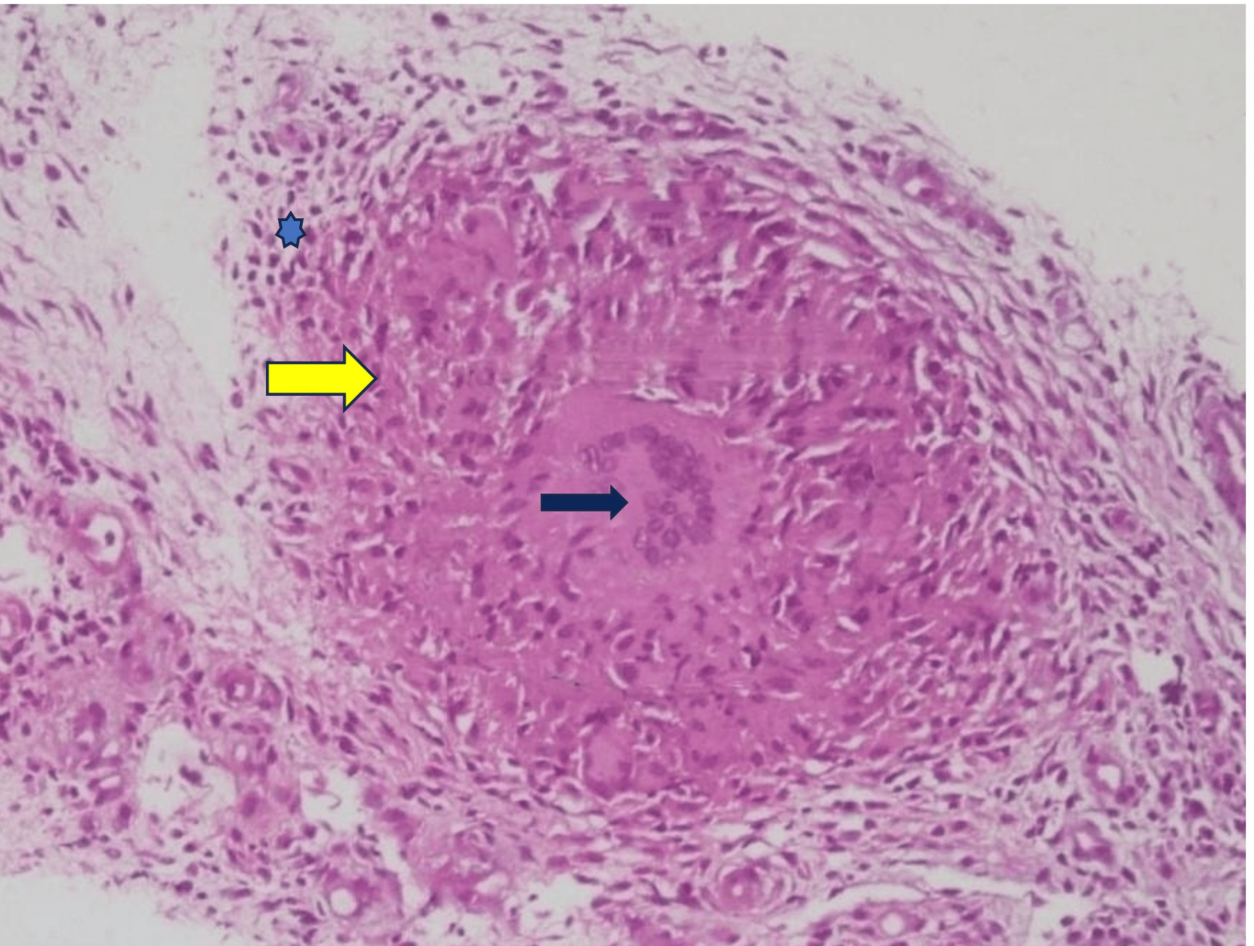
Microscopic examination of the synovial tissue showing granuloma (H&E staining) The yellow arrow indicates a granuloma, the black arrow shows a giant cell, and the blue star indicates a chronic inflammatory cell. Photograph courtesy of the Department of Pathology, Bangladesh Medical University (BMU).

USG of the left knee joint showed joint effusion with synovial proliferation in Figure 11 and a ruptured Baker cyst. The synovial fluid study was negative for AFB and Gram stain, MT, and HLA-B27 were negative. A synovial biopsy from the left knee joint was done. It revealed chronic inflammatory cells with granuloma, and in the center of the granuloma, a giant cell was present (Figure 12).

His condition had improved gradually after taking anti-tubercular treatment during the one-month follow-up. Sociodemographic and laboratory findings of this case series on tubercular arthritis are shown in Tables 1-2.

**Table 1:** Laboratory findings of this case series on tubercular arthritis SB = synovial biopsy, DM= diabetes mellitus, SF = synovial fluid, MT = Mantoux test, AFB = acid-fast bacilli

Parameter with unit	Case 1	Case 2	Case 3	Case4	Case 5	Normal value
Hb%, gm /dl	11	7.6	10	11	10	12-18
ESR mm in the first 1 hour	15	60	50	60	58	<10
WBC/cu mm of blood	5500	3200	4500	5000	17	4000-11000
Neutrophil, %	60	50	55	57	50	40-80
Lymphocyte, %,	30	47	45	44	45	20-40
Eosinophil, %	6	1	4	3	3	1-5
Monocyte, %	2	0	2	2	1	2-10
Basophil, %	2	4	2	3	2	<1
CRP, mg/dl	13	12	15	20	20	<6
MT in mm after 72 hours	15	15	8	18	8	up to 10
RBS, mmol/dl	4.4	6	7	8	6	<11.1
SGPT, U/l	19	20	30	40	50	<40
Creatinine, mg/dl	0.91	0.70	0.9	1.0	1.1	1.2-1.4
Xray finding	Calcaneal spar	Soft tissue swelling	Soft tissue swelling	Soft tissue swelling	Soft tissue swelling	Normal
US finding	Synovial hypertrophy with grade 2 Doppler	Synovial hypertrophy	Synovial hypertrophy	Synovial hypertrophy with fluid in rt knee joint with grade 1 Doppler	Effusion with synovial proliferation& ruptured Baker cyst	Normal
SF cytology/cuml	Not done	Not done	Not done	120,000/cu ml	Not done	<50
SF AFB staining	Not found	Not found	Not found	Positive	Not found	No AFB
SF-GeneXpert	Not found	Negative	Negative	Positive	Positive	Negative
SB finding	Multiple epithelioid-type granulomas surrounded by lymphocytes	Caseation necrosis and granuloma with inflammatory cells	Multiple epithelioid-type granulomas with inflammatory cells	Caseation necrosis and granuloma with chronic inflammatory cells	Infiltration of chronic inflammatory cells with caseation necrosis	
SB GeneXpert	Negative	Negative	Negative	Negative	Negative	Negative
Comorbid disease	DM	No	No	DM	No	-

**Table 2:** Sociodemographic variables of this case series on tubercular arthritis

Variable	Case 1	Case 2	Case 3	Case4	Case 5
Age/Sex	50/M	17/F	50/M	45/M	60/M
Occupation	Cultivator	Garments worker	Cultivator	Cultivator	Businessman
Economic status	Low	low	Low	low	High
Duration of symptoms	Left ankle pain, 5 months	Rt ankle, 1 month	Left ankle, 6 months	RT knee, 1.5 years	Oligoarthritis 6 months
Systemic feature	No	Present	Present	Present	No
Previous history	No	No	No	No	Spondyloarthritis
Active & passive movement	Restricted	Restricted	Restricted	Restricted	Restricted
USG finding	Synovial hypertrophy	Synovial hypertrophy	Synovial hypertrophy	Synovial hypertrophy	Synovial hypertrophy

## Discussion

It is reported that 10% of cases of tuberculosis affect a single joint. It may affect multiple joints [4,5]. The hip is the most common joint, followed by the knee joints, sacroiliac joint (SIJ), and shoulder, elbow, and ankle joints [6]. Thirty percent of cases experience fever and weight loss [7]. The hip joint is deep-seated and difficult to diagnose by the usual method [8]. USG-guided synovial biopsy (USG SB) was done for histopathology with GeneXpert. USG findings of the joint were synovial hypertrophy with grade 1 to 2 Doppler; however, MRI is sensitive for tuberculous arthritis but not specific. The MRI features of tuberculous arthritis include bone marrow edema, cortical erosion, synovitis, joint effusion, tendon synovitis, soft tissue involvement, and myositis; however, this could not be performed due to financial constrain [5,9]. All five patients underwent joint USG, which showed synovitis and joint effusion, and underwent a synovial biopsy; biopsy is essential for diagnosing tuberculous arthritis. Positive histological findings of tuberculosis were found in 64-90% of patients [10,11]. In our case series, a synovial biopsy was performed for all patients, and they were diagnosed with tuberculous arthritis.

Immunocompromised patients, HIV infection, malnutrition, long-term immunosuppressive treatment, malignancies, renal failure, extreme age, and organ transplantation are risk factors for skeletal tuberculosis [12,13]. Among the five patients in this series, two patients had diabetic mellitus.

Tubercular arthritis may present with pain, swelling, and movement restrictions [14]. Traumatic arthritis, pyogenic arthritis, rheumatoid arthritis, psoriatic arthropathy, sarcoidosis, and neoplasms are the differential diagnoses for tubercular arthritis [14].

Atypical presentations frequently cause diagnostic challenges. Luke et al. reported in 2017 that isolated joint involvement by tubercular arthritis is rare in immunocompetent patients [15]. In our case series, there was no history of tuberculosis or immunocompromised status. The sensitivity of acid-fast bacilli in extrapulmonary TB is 45-80%. Elbrolosy et al. reported that the sensitivity and specificity of GeneXpert for extrapulmonary TB and the microscopic findings of acid-fast bacilli (AFB) are 81.6% and 78.9%, and 63.2% and 70.5%, respectively [16]. Wen et al. reported that the sensitivity and specificity of GeneXpert in joint TB were 81% and 83%, respectively [17]. GeneXpert is a test for both pulmonary and extrapulmonary TB, requiring only two hours. It is recommended by the World Health Organization (WHO) for adults, children, and HIV and non-HIV patients [2]. The rates of AFB in synovial fluid are very low (10-20%) [18].

## Conclusions

Tuberculosis remains one of the most common causes of undifferentiated monoarthritis, especially in endemic regions. Elevated ESR and CRP levels can support the suspicion of tubercular arthritis. A definitive diagnosis is made through a synovial biopsy. An ultrasound-guided synovial biopsy, along with GeneXpert testing, with or without AFB staining, of synovial fluid or tissue, is an essential diagnostic tool. A high index of clinical suspicion is crucial for the timely diagnosis and initiation of treatment to prevent irreversible joint damage.
